# Risk factors and multi-pathogen infections in kidney transplant recipients with omicron variant pneumonia: a retrospective analysis

**DOI:** 10.1186/s12879-024-09444-4

**Published:** 2024-06-04

**Authors:** Jing Chen, Yuanbo Su, Ming Lu

**Affiliations:** 1 Department of Infectious Disease, Peking University Third Hospital, Beijing, 100191 China; 2 Department of Respiratory and Critical Care Medicine, Peking University Third Hospital, Beijing, 100191 China; 3 Infectious Disease Center, Peking University Third Hospital, Beijing, 100191 China

**Keywords:** Kidney transplant recipients, COVID-19 virus variant Omicron, Coinfections, Risk factors

## Abstract

**Background:**

Kidney transplant recipients (KTRs) are at an elevated risk of progressing to severe infections upon contracting COVID-19. We conducted a study on risk factors and multi-pathogen infections in KTRs with SARS-CoV-2 Omicron variant.

**Methods:**

KTRs were subjected to a thorough etiological evaluation. Whenever feasible, they were also provided with bronchoscopy and bronchoalveolar lavage to enable metagenomic next-generation sequencing (mNGS), ideally within a 48-hour window post-admission. We performed a retrospective analysis for pathogens and risk factors of KTRs with the COVID-19 virus variant Omicron.

**Results:**

We included thirty patients in our study, with sixteen exhibiting single infection of COVID-19 and fourteen experiencing co-infections, predominantly with Pneumocystis jirovecii. Notably, patients with severe cases demonstrated significantly elevated levels of C-reactive protein (CRP) and interleukin-6 compared to those with moderate cases (*P* < 0.05). Furthermore, individuals whose conditions progressed had markedly higher baseline serum creatinine levels than those without such progression (*P* < 0.05). The presence of heart failure, acute exacerbation of renal dysfunction, and a history of opportunistic infections were significantly associated with a higher likelihood of deterioration and hospital admission due to the SARS-CoV-2 Omicron variant, as compared to the control group (*P* < 0.05). In subsequent follow-up analysis, the all-cause rehospitalization rate was observed to be 21.4%, with Pneumocystis jirovecii infection accounting for half of these cases.

**Conclusion:**

Among KTRs, a significant coinfection rate of 47% was observed, with Pneumocystis jirovecii emerging as the predominant pathogen in these cases. The development of heart failure, acute exacerbation of chronic renal dysfunction, and a prior history of opportunistic infections have been identified as potential risk factors that may contribute to clinical deterioration in KTRs. Additionally, Pneumocystis jirovecii infection has been established as a critical factor influencing the rate of all-cause rehospitalization within this patient population.

## Background

Severe acute respiratory syndrome coronavirus 2 (SARS-CoV-2) is a highly transmissible and pathogenic coronavirus that emerged in late 2019 and has caused a pandemic of acute respiratory disease-coronavirus disease 2019(COVID-19), and over 767 million confirmed cases have been reported to the World Health Organization (WHO), including approximately 6.94 million deaths. Although most infections in the general population are mild and have a good prognosis, kidney transplant recipients have poorer prognosis. The immunosuppressed state makes them highly susceptible to severe COVID-19 infections and increases their mortality rates [1]. Furthermore, kidney transplant recipients are also prone to opportunistic infections, such as Pneumocystis jirovecii and cytomegalovirus, which can be difficult to distinguish from COVID-19 pneumonia. In December 2022, the SARS-CoV-2 Omicron variant pandemic significantly impacted the Chinese population, with kidney transplant patients being particularly vulnerable. To address this, we undertook a study specifically examining the risk factors and multi-pathogen infections associated with Omicron variant pneumonia in KTRs. This research diverges from previous studies that focused on multiple subtypes of COVID-19. Notably, our study predominantly utilized Bronchoalveolar Lavage Fluid (BALF) for mNGS, marking the first comprehensive investigation of coinfection pathogens in KTRs with the Omicron variant using this approach.

## Methods

### Study design

A retrospective analysis was conducted on kidney transplant recipients with omicron variant. The research protocol was approved by the Ethics Committee of the Peking University Third Hospital [Ethical Approval Number: 2021(214-01)], and informed consent was obtained from all participants.

### Study subjects

Patients with postkidney transplantation and subsequent COVID-19 infection admitted to the Infectious Diseases Department of Peking University Third Hospital between January 1st and August 31st 2022 were enrolled in this cohort study. The inclusion criteria were (1) aged ≥ 18 years; (2) post kidney transplantation; (3) met the diagnostic criteria of New Coronavirus Infection Diagnosis and Treatment Plan (China Trial Version 10) [2]. (4) The SARS-CoV-2 strain is identified as Omicron variant by Sanger sequence or BALF-mNGS. (5) Ethnicity: Asian Chinese Han population.

The exclusion criteria were as follows: patients with other diseases that cause immunosuppression, including malignancy patients undergoing chemotherapy, transplantation of organs other than kidneys, hematopoietic stem cell transplantation, and HIV infections, or other autoimmune diseases. We meticulously reviewed the medical records of KTRs to obtain detailed information, particularly regarding their prior history of opportunistic infections. Additionally, we conducted a thorough inquiry into the patients’ previous treatments and any documented opportunistic infections.

**Fig. 1:**
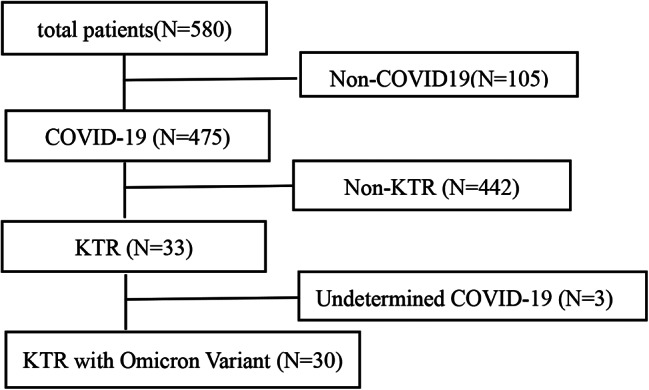
Flowchart of patient selection proces in the study

Over the course of our research period, we admitted a total of 580 patients. Among these, 475 were identified with COVID-19. Within this group, we identified 33 KTRs. Of these KTRs, 30 were confirmed to have the Omicron variant, while the remaining 3 had COVID-19 cases that not be definitively classified. Ultimately, it was these 30 KTRs with the Omicron variant who were included in the final analysis (Fig. 1).

### Data collection

Data were collected on the demographic characteristics of all patients, including age and sex, medical history, physical examination, laboratory tests, chest CT scans, treatment plans, and clinical outcomes.

### Specimen collection and pathological testing

Upon admission, within a 48-hour window, we systematically collected a range of diagnostic samples. This included blood cultures, sputum cultures, and smear microscopy with specific attention to acid-fast staining. We performed sputum multipathogen PCR testing to identify a spectrum of bacterial and viral pathogens, including Streptococcus pneumoniae, Staphylococcus aureus, Klebsiella pneumoniae, Pseudomonas aeruginosa, Haemophilus influenzae, Acinetobacter baumannii, Burkholderia cepacia, as well as influenza virus, respiratory syncytial virus, parainfluenza virus, adenovirus, Mycoplasma, Chlamydia, and Legionella pneumophila.

Furthermore, whole blood multipathogen PCR testing was predominantly employed for the quantitative detection of human cytomegalovirus and Epstein-Barr virus. In addition to PCR-based methods, we also conducted blood tests for 1,3-β-D-glucan (G-test) and galactomannan antigen (GM test) to enhance the diagnostic accuracy for fungal infections. If permitted by the patients’ consent and their medical condition, bronchoscopy and bronchoalveolar lavage were conducted within a 48-hour window following hospital admission.BALF was submitted for mNGS. If lung inflammation was diffusely distributed with a uniform morphology (such as widespread ground-glass opacity), bronchoalveolar lavage was typically performed in the right middle lobe or lingular segment of the left upper lobe. If lung inflammation was present in multiple lobes with varied morphologies (including ground-glass opacities, consolidation, and nodules), multiple-point lavages were conducted across different lung lobes (with a total lavage volume not exceeding 200 ml). BALF samples collected from different lobes were mixed and promptly sent for NGS analysis. The patients who did not get BALF- mNGS would get SARS-CoV-2 Sanger sequencing for the strain confirmation.

Patients are categorized into mild, moderate, severe, and critical types of COVID-19 based on the classification criteria outlined in the New Coronavirus Infection Diagnosis and Treatment Plan (China Trial Version 10) [2]. 

### Statistical analysis

Statistical analysis was conducted using SPSS software version 20.0. Categorical data are expressed as frequencies and percentages. Normally distributed continuous data are represented as the mean ± standard deviation, while nonnormally distributed data are expressed as medians with interquartile ranges. T tests were applied to normally distributed data, whereas nonparametric tests or chi-square tests were used for nonnormally distributed data. A P value of less than 0.05 indicated a statistically significant difference.

## Results

### Clinical characteristics of KTRs in our study

Thirty patients were included in the study. The median age was 49.5 years (IQR: 43.5–56.3 years). Twenty-five were male, and five were female. The median duration since kidney transplant was 84.5 months (IQR: 31.8-120.0months). None of the 30 patients had a history of receiving the COVID-19 vaccine. The condition of 5 patients (13.3%) was progressive during their hospital stay (Table 1). On average, the patients experienced 12.0 days (IQR: 8.5–20.0 days) of illness before admission. Twenty-one patients (70%) were diagnosed with a moderate form of COVID-19, and 9 patients (30%) were diagnosed with the severe form (Table 1).

**Table 1:** Clinical characteristics of the KTRs

	*N* = 30
Age(years)	49.5(43.5–56.3)
Gender(Male/Female)	25/5
Time since Kidney Transplant(months)	84.5(31.8–120.0)
Time before Hospital Admission(days)	12.0(8.5–20.0)
**Clinical Classification**	
Moderate	*N* = 21(70%)
Severe	*N* = 9(30%)
**Treatment**	
Corticosteroids	*N* = 30(100%)
Antiviral treatment Nirmatrelvir/Ritonavir Molnupiravir	*N* = 14(46.7%) *N* = 16(53.3%)
Tocilizumab Baricitinib	*N* = 9(30%) *N* = 2(6.7%)
**Clinical Outcome**	
Disease progression (developed into severe/critical type/die)	*N* = 5
Intubation	*N* = 2
Transplanted kidney dysfunction	*N* = 3
Death	*N* = 2
**BALF + mNGS**	
YES	27
NO	3
Infection	
single COVID19 infection	*N* = 16
COVID19 + bacteria	*N* = 3
COVID19 + PJP	*N* = 3
COVID19 + PJP + CMV	*N* = 2
COVID19 + PJP + bacteria	*N* = 2
COVID19 + PJP + Aspergillus	*N* = 1
COVID19 + CMV + Aspergillus	*N* = 2
COVID19 + PJP + CMV + Aspergillus	*N* = 1

### Microbiological findings

**Fig. 2:**
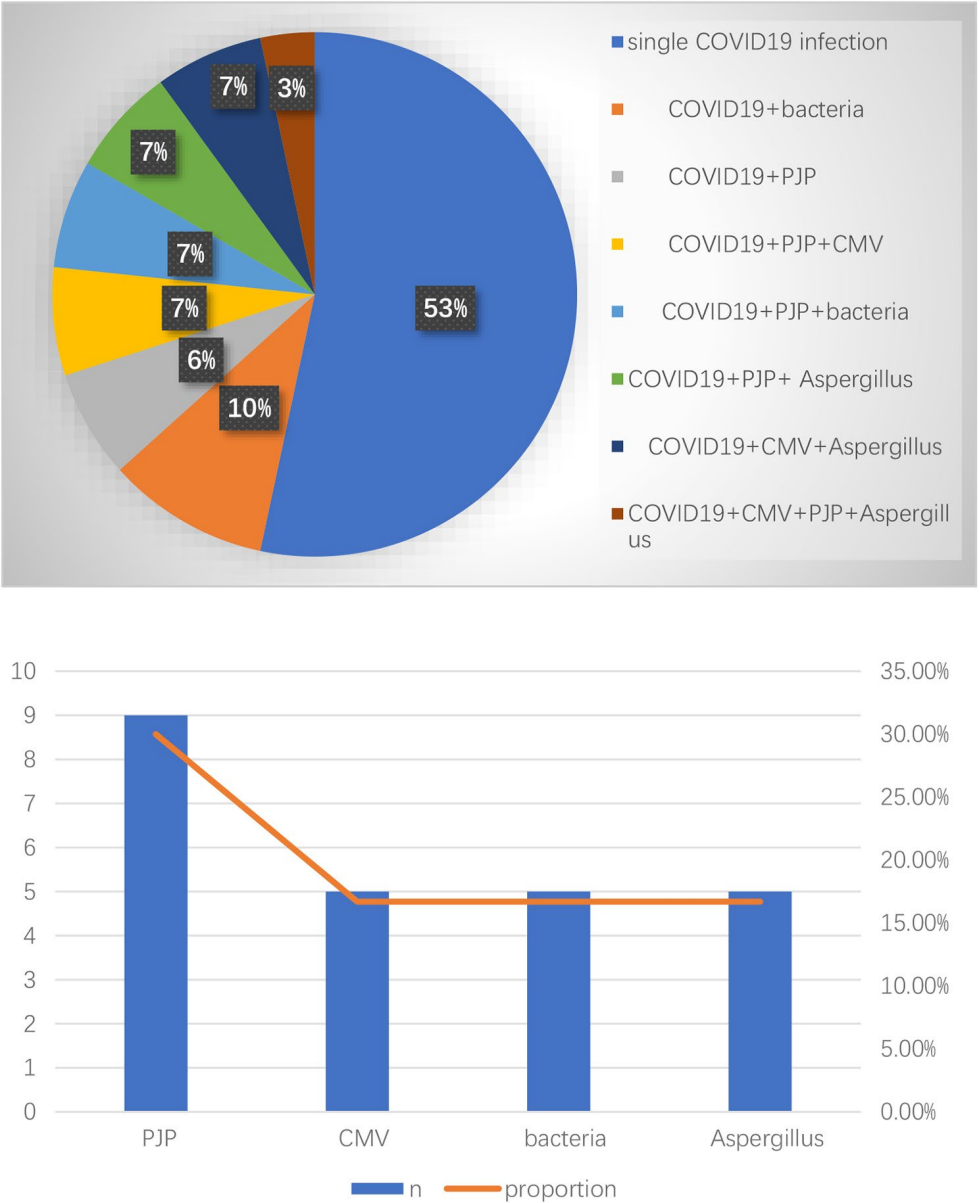
**a** Pathogens coinfected in kidney transplant patients with novel coronavirus infection. **b** Specific distribution of coinfections in kidney transplant patients with novel coronavirus infection

Out of the 30 patients in our study, BALF was subjected to mNGS in 27 cases. The remaining 3 patients did not undergo bronchoscopy. Despite this, routine microbiological assessments failed to yield any positive indications of coinfection. Our analysis showed that 16 patients experienced a singular infection with the COVID-19 virus, whereas 14 patients had coinfections involving the COVID-19 virus and other pathogens, as illustrated in Fig. 2.

- Nine had concurrent infections with Pneumocystis jirovecii.
- Five had bacterial coinfections: Klebsiella pneumoniae (*N* = 3), Staphylococcus aureus(*N* = 1), Proteus mirabilis (*N* = 1).
- Five had cytomegalovirus infection.
- Five had aspergillosis infections.

As shown in Fig. 2; Table 1, among the KTRs, 53% experienced a single COVID-19 infection. The rate of co-infection was 47%. The most prevalent co-infection was Pneumocystis jirovecii followed by cytomegalovirus , bacterial infections, and aspergillosis.

### Treatment and prognosis

In our cohort of 30 patients, 21 (70%) were diagnosed with a moderate form of COVID-19, while 9 (30%) presented with severe cases, as classified by the New Coronavirus Infection Diagnosis and Treatment Plan (China Trial Version 10). Post-hospital admission, we observed a loss of transplanted kidney function in 3 patients. Additionally, 3 patients with critical conditions required mechanical ventilation. Notably, BALF analysis in one of the critical patients indicated a concurrent infection with Pneumocystis jirovecii and Aspergillus. Another critical patient was found to have a concurrent infection with Klebsiella pneumoniae.

All patients were treated with antiviral therapy (/Nirmatrelvir/Ritonavir or Molnupiravir) and corticosteroids postadmission (Table 1).

- Nine patients (30%) received tocilizumab, and two patients (6.7%) were administered baricitinib.
- Nine patients with concurrent Pneumocystis jirovecii infection were treated with high doses of sulfonamide.
- Five patients with cytomegalovirus coinfections were given ganciclovir.
- Five patients underwent antifungal treatment with voriconazole, targeting Aspergillus infection.

### Subgroup analysis based on clinical typing of COVID-19 at admission

The 30 patients were divided into two groups based on the clinical type of COVID-19 infection: the moderate type group (*N* = 21) and the severe type group (*N* = 9). A nonparametric test was used to compare the two groups (Tables 2 and 3).

**Table 2:** Chi-square test analysis based on clinical classifications, coinfections, and disease progression

	Group1	Group2	*X* ^2^	*P*
	Moderate Group *N* = 21	Severe Group *N* = 9		
Usage of antiviral drugs before hospitalization			1.59	0.71
No	14	8		
Yes	7	1		
Coinfections			0.92	0.34
No	10	6		
Yes	11	3		
	No coinfection *N* = 14	Coinfection *N* = 16		
Previous opportunistic infection status			0.40	0.53
No	13	10		
Yes	3	4		
Usage of antiviral drugs before hospitalization			0.05	0.83
No	12	10		
Yes	4	4		
	Stable condition *N* = 25	Worsened condition *N* = 5		
Previous opportunistic infections			4.51	0.034*
No	21	2		
Yes	4	3		
Coinfection analysis			0.43	051
No	14	2		
Yes	11	3		
Heart failure			5.23	0.002*
No	25	3		
Yes	0	2		
Renal dysfunction			17.33	0.000*
No	24	1		
Yes	1	4		
Covid-19 clinical typing			0.29	0.60
Moderate	17	4		
Severe	8	1		
Antiviral medications prior to hospital admission			0.14	0.71
No	18	4		
Yes	7	1		

**Table 3:** The clinical features and prognoses of KTRs

	Gender	Age(years)	Time since Kidney Transplant(months)	BALF	co-infection status	moderate/severe grouping	stable/worsening grouping	Heart/ kidney function	IL-6 baseline	CRP baseline	prognoses
1	M	54	94	YES	COVID-19	moderate	worsening	/	7.9	13.3	gastrointestinal bleeding
2	M	32	85	YES	COVID19 + PJP	moderate	worsening	Transplanted kidney dysfunction	9.5	109.9	hematodialysis
3	M	37	60	YES	COVID19 + CMV + PJP	Moderate	stable	/	7.2	48.6	same as before covid-19
4	M	33	168	YES	COVID-19	severe	stable	/	51.3	78.5	same as before covid-19
5	F	54	110	YES	COVID-19	moderate	stable	/	10.2	1.7	same as before covid-19
6	M	35	42	YES	COVID-19	moderate	stable	/	13.4	9.8	same as before covid-19
7	F	46	120	YES	COVID-19	moderate	stable	/	18.3	6.8	same as before covid-19
8	M	52	36	YES	COVID-19	severe	stable	/	209.0	21.7	same as before covid-19
9	M	56	108	YES	COVID19 + CMV + Aspergillosis	severe	worsening	heart failure. kidney dysfunction	21.8	113.5	same as before covid-19
10	M	44	108	YES	COVID-19 + Klebsiella pneumoniae	moderate	stable	/	26.7	84.6	same as before covid-19
11	M	60	132	YES	COVID-19	moderate	stable	/	12.5	8.0	same as before covid-19
12	M	45	12	YES	COVID-19 + Staphylococcus aureus	moderate	stable	/	14.1	23.8	same as before covid-19
13	M	57	264	YES	COVID19 + PJP + Klebsiella pneumoniae	moderate	stable	/	12.3	40.3	same as before covid-19
14	M	31	120	YES	COVID-19	moderate	stable	/	11.3	9.9	same as before covid-19
15	M	53	2	YES	COVID19 + CMV + PJP	moderate	stable	/	176.0	60.1	same as before covid-19
16	M	47	84	YES	COVID19 + PJP + CMV + Aspergillosis	moderate	stable	kidney dysfunction	2.2	25.7	Transplanted kidney dysfunction-hematodialysis. and heart failure for twice hospitalized
17	M	50	108	YES	COVID19	severe	stable	/	30.0	70.1	PJP
18	M	50	48	YES	COVID19 + PJP + Aspergillosis	severe	worsening (intubation,,death)	heart failure. kidney dysfunction	10.2	31.7	/
19	M	67	4	YES	COVID19 + CMV + Aspergillosis	moderate	stable	/	7.0	6.6	same as before covid-19
20	F	70	19	NO	COVID19	severe	stable	/	63.6	128.6	PJP
21	M	75	240	NO	COVID19	severe	stable	/	1187.0	51.1	same as before covid-19
22	M	31	48	YES	COVID19 + PJP + Aspergillosis	moderate	stable	/	5.7	23.0	same as before covid-19
23	M	55	12	YES	COVID19 + CMV	moderate	stable	/	5.7	10.4	same as before covid-19
24	F	64	156	NO	COVID19	moderate	stable	/	5.4	51.4	same as before covid-19
25	F	42	48	YES	COVID19	moderate	stable	/	22.6	70.9	same as before covid-19
26	M	49	12	YES	COVID19	severe	stable	/	9.4	75.0	PJP
27	F	47	78	YES	COVID19 + Proteus mirabilis	severe	stable	/	3.5	119.0	same as before covid-19
28	M	68	132	YES	COVID19	moderate	stable	/	10.1	69.9	same as before covid-19
29	M	48	1	YES	COVID19 + Klebsiella pneumoniae	moderate	worsening (intubation, death)	kidney dysfunction	9.7	71.0	/
30	M	54	94	YES	COVID19	stable	/	stable	224.0	74.7	same as before covid-19

#### Biomarkers

Patients in the severe group had significantly higher levels of CRPand interleukin-6 (IL-6) than those in the moderate group. This difference was statistically significant (*P* < 0.05). No significant differences were observed in other clinical indicators between the two groups (Table 4).

**Table 4:** Clinical characteristics of KTR by subgroup analysis

	Clinical Classification		*P*	COVID19 co-infections		*P*	disease progression after admission		*P*
	Moderate Group	Severe Group		No co-infection	co-infection		Stable condition group	Worsened group	
Age(years)	48.0(39.5–56.0)	50.0(47.0–63.0)	0.37	51.0(43.0–63.0)	47.5(42.3–55.3)	0.40	49.0(43.0-58.5)	50.0(40.0–55.0)	0.10
Time since Kidney Transplant(months)	84.00(27.00-120.00)	96.00(27.50–138.00)	0.67	103.00(43.50–132.00)	68.00(10.00-108.00)	0.08	84.00(27.5–126.00)	85.00(24.50–101.00)	0.49
Time before Hospital Admission(days)	12.00(7.00–20.00)	12.00(10.50–20.00)	0.65	10.25(14.00–20.00)	6.75(10.50–20.00)	0.35	14.00(10.00–20.00)	9.00(3.50-9.00)	0.04*
BMI (kg/m^2^)	23.12(21.25–24.24)	26.12(20.13–28.50)	0.22	24.00(21.46–26.36)	22.99(20.48–26.69)	0.79	23.84(21.55–25.64)	23.12(19.59–32.19)	0.80
white blood cell	6.25(3.68–8.35)	5.17(3.83–8.78)	0.91	5.76(3.96–7.35)	6.60(3.68–8.64)	0.59	6.00(3.83–8.35)	6.94(3.07–9.05)	0.98
leukomonocyte	0.60(0.32–0.85)	0.59(0.32–0.74)	0.68	0.61(0.35–0.91)	0.57(0.31–0.77)	0.48	0.62(0.34–0.83)	0.29(0.22–1.06)	0.78
neutrophil	4.64(2.73–7.26)	4.27(2.71–8.53)	0.64	4.29(2.75–6.64)	5.30(2.70–7.85)	0.32	4.37(2.72–7.33)	5.26(2.44–8.26)	0.90
monocyte	0.32(0.18–0.51)	0.32(0.27–0.43)	0.86	0.37(0.23–0.49)	0.30(0.25–0.50)	0.56	0.32(0.27–0.49)	0.37(0.11–0.51)	0.6
platelet	172.00(121.50–235.00)	159.00(92.50-183.50)	0.20	159.00(96.25–192.00)	167.509123.25–215.50)	0.39	167.00(115.50–214.00)	164.00(116.50–187.00)	0.74
C-reactive protein*	25.70 (9.85-65.00)	75.00(60.50-116.25)	0.04*	41.40(9.82–73.75)	54.35(23.60-90.92)	0.32	48.60(10.15–72.86)	71.00(22.50-111.70)	0.25
Procalcitonin	0.12(0.07–0.19)	0.25(0.07–0.98)	0.17	0.14(0.05–0.24)	0.14(0.07–0.42)	0.55	0.12(0.06–0.21)	0.20(0.13–1.77)	0.06
Interleukin 6*	10.05(7.03–14.08)	51.30(15.62–216.50)	0.02	15.82(9.89–15.82)	9.60(5.69–23.02)	0.07	13.38(7.03–51.30)	8.74(7.95–21.8)	0.49
ferritin	614.50(278.75–810.50)	662.00(421.00-1307.00)	0.42	642.00(15.82–789.00)	607.00 (269.50–945.00)	0.83	604.5(299.50-812.75)	674.00(476.50–1153.00)	0.36
ALT	14.00(9.40–32.00)	29.00(13.35–58.70)	0.12	21.50(9.50–30.50)	14.85(9.88–43.75)	0.93	16.80(9.95-30.00)	35.00(7.00-51.50)	0.72
AST	23.00(15.05-29.00)	27.00(16.20–68.50)	0.29	23.00(14.90–40.30)	23.50(15.03–31.50)	0.97	14.95(23.00-30.50)	30.00(16.25-53.00)	0.45
LDH	243.0(218.00-305.00)	276.00(223.50–453.00)	0.29	235.00(219.24–273.50)	289.0(221.75–458.50)	0.14	255.00(217.50–289.00)	327.00(231.0-517.50)	0.17
TBIL	8.40(4.75–14.75)	6.90(5.30-10.15)	0.35	7.95(5.90-16.83)	8.50(4.45–11.25)	0.69	7.90(5.10-11.65)	10.7 (5.85–14.7	0.47
CRE	121.0(102.00-211.00)	147.0(95.0-246.00)	0.82	123.50(104.75-221.25)	139.50(95.25–224.50)	0.98	116.00(99.50–179.00)	312(172.50-326.50)	0.01*
UREA	9.55(7.27–13.05)	11.30(8.15–23.40)	0.42	9.05(7.47–11.70)	11.44(7.31–18.48)	0.32	9.68(5.90–46.3)	12.90(9.45–33.35)	0.15
CD4-T cell	139.00(82.84-283.43)	186.60(66.40-344.50)	0.78	274.00(77.32–393.00)	115.75(79.51–164.00)	0.08	164.80(84.44–314.00)	70.00(46.75.-161.50)	0.09
CD8-T cell	179.73(134.15-319.75)	183.00(72.75–264.40)	0.51	230.00(92.00-425.00)	163.66(118.21-262.85)	0.18	199.50(138.78–297.50)	92.00(49.20-317.50)	0.30
CD19-B cell	33.33(17.82–50.67)	48.00(30.75–74.86)	0.24	32.65(44.82-66.00)	27.29(16.94–55.75)	0.19	43.41(17.82–68.04)	32.65(25.50–38.80)	0.50

*p < 0.05

These findings suggest that biomarkers such as CRP and IL-6 can indicate the severity of COVID-19 in KTRs.

### Subgroup analysis based on the presence of coinfections beyond the novel coronavirus

The 30 cases were divided into two groups based on the presence of coinfection: single COVID-19 virus variant Omicron infection (*N* = 16) and coinfections (*N* = 14). Nonparametric statistical analysis revealed no significant differences between the two groups. An assessment of the history of opportunistic infections and antiviral treatments administered prior to hospitalization using the chi-square test also showed no significant differences (*P* > 0.05) (Tables 2 and 3).

### Subgroup analysis of predictive factors for deterioration post-admission

**Fig. 3:**
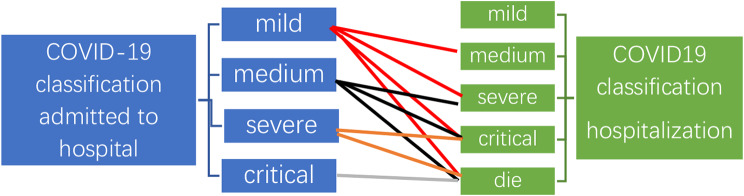
The description of progressing group: COVID-19 classification progress from admitted to hospital to hospitalization

In our study, we defined the “progressive group” as those patients whose COVID-19 classification worsened from the baseline type to a more severe category following hospitalization. This progression could range from mild to moderate, severe, critical, or even fatal, as depicted in Figs. 3 . Conversely, patients whose COVID-19 classification remained the same or improved post-hospitalization were categorized as the “stable group”, as shown in Fig. 3.

**Fig. 4:**
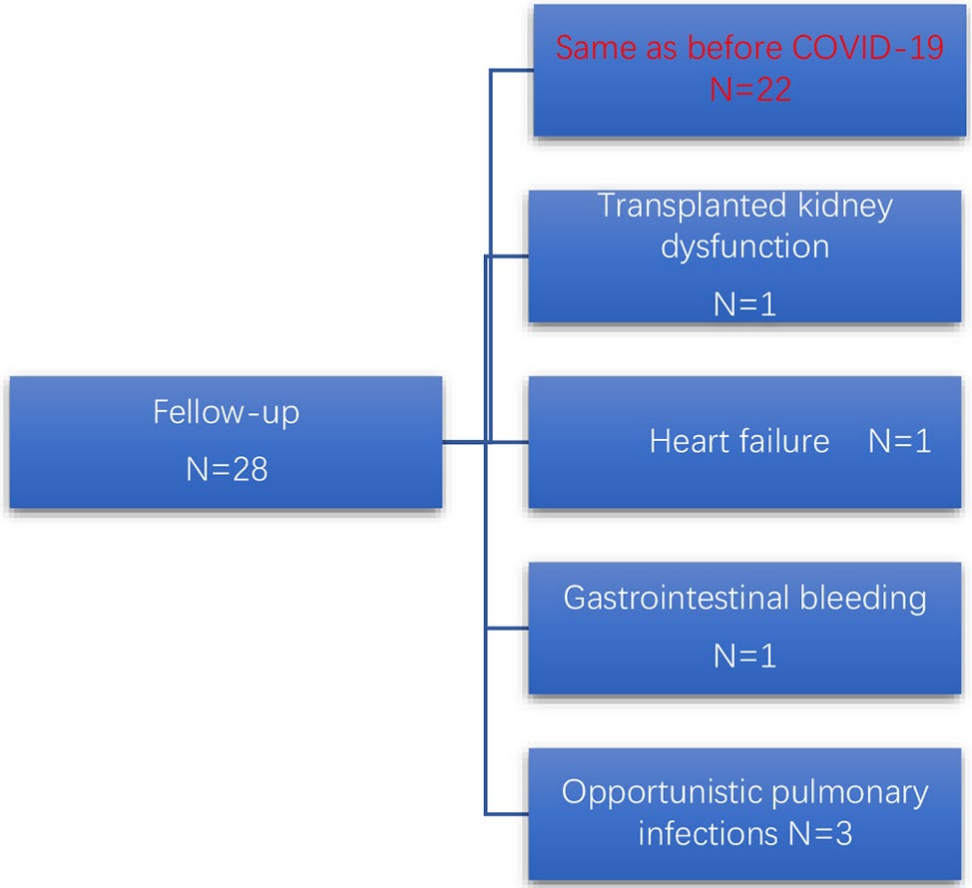
Follow-up of kidney transplant patients with COVID-19 conditions after discharge

The cohort was divided into two groups: the stable group, comprising 25 patients, and the progressive group, with 5 patients. This categorization was based on the observed conditions during hospitalization, such as a shift from moderate to severe or critically severe stages. Our analysis revealed significantly higher baseline creatinine levels in the progressive group compared to the stable group (*P* < 0.05). No significant differences were observed in other clinical indicators between these two groups.

We conducted statistical analyses using chi-square tests to assess potential predictive factors for post-admission deterioration in KTRs. These factors included a history of opportunistic infections, presence of coinfections, concurrent heart failure, acute exacerbation of renal dysfunction, and the clinical type of COVID-19 pneumonia upon admission. The results underscored that heart failure, acute exacerbation of renal dysfunction, and a history of opportunistic infections were significant risk factors for deterioration post-hospitalization in KTRs, as detailed in Tables 2 and 3.

### Follow-up analysis

As shown in Fig. 3, over a follow-up period of 4.5(2.0–7.0) months, the all-cause rehospitalization rate was 21.4% (6/28). Rehospitalization reasons were heart failure (*N* = 1), transplant kidney dysfunction (*N* = 1), gastrointestinal bleeding (*N* = 1), and pulmonary opportunistic infections (Pneumocystis jirovecii *N* = 3). No cytomegalovirus or Aspergillus infections were observed (Fig. 3).

## Discussion

Kidney transplant recipients are prone to opportunistic infections, such as Pneumocystis jirovecii and cytomegalovirus infections, due to prolonged immunosuppression and susceptibility to a variety of pathogens. KTRs have a poorer prognosis than the general population with regard to COVID-19 infections. Early data revealed that the mortality rate of KTRs infected with COVID-19 ranges from 20 to 32% [1, 3–5], according to data from WHO, the infection fatality ratio for COVID-19 in the general population is estimated to converge at approximately 0.5 to 1% [6].

In multiple early reviews concerning postkidney transplant COVID-19 infections, the occurrence of severe complications such as respiratory distress, acute kidney injury, and acute myocardial infarction, as well as the rate of severe cases and mortality, were significantly higher than in the general population [7, 8]. The previous studies were based on multiple variants of COVID-19 (including alpha, beta, delta, etc.) Our study is solely focused on the variant Omicron, which was a pandemic in China in 2023 and gave us a real prognosis for the COVID-19 virus variant Omicron in KTRs.

In our analysis, both heart and kidney dysfunction, as well as a history of opportunistic infections, significantly influenced the exacerbation of COVID-19 posthospitalization. Patients with deteriorating heart and kidney function should be particularly risk factors for disease progression. The severity categorization of COVID-19 in kidney transplant patients and concurrent infections does not contribute as risk factors for posthospitalization exacerbation.

KTRs experience rapid disease progression after COVID-19 infection. A multicenter study in Italy in 2020 found that approximately 75% of patients experienced acute exacerbation within one week, 39% needed mechanical ventilation, and 21% needed renal replacement therapy [9]. The outcomes varied greatly for the different research times, viral strains and countries. In our study, all patients were infected with the Omicron strain of SARS-CoV-2 in 2023. The included data cover hospitalizations from January ^1st^ to August ^31st,^ 2023, with access to advanced life support conditions such as ICU admission, mechanical ventilation, and ECMO, accurately reflecting the outcomes of moderate and severe cases postkidney transplant with Omicron variant infections. In our study, the mortality rate was 6.7%, which is lower compared with earlier data on mortality rates in postkidney transplant COVID-19 patients.

Furthermore, our study revealed that KTRs had a high rate of coinfection with coronavirus and other pathogens, reaching up to 46.6%. Pathogens were Pneumocystis jirovecii (*N* = 9, 56.3%), bacteria (*N* = 5, 16.7%), cytomegalovirus (*N* = 5, 16.7%), and aspergillosis (*N* = 5, 16.7%). A multipathogeninfection was identified (*N* = 7, 23.3%). Both our clinical experience and the literature suggest that the general population rarely experiences coinfections in the early stages of COVID-19, with coexisting bacterial infections seen in approximately 3–8% of the general COVID-19-positive population. Among those with severe forms, approximately 12% experience bacterial infections, and the prevalence of mixed bacterial and fungal infections is 8%. Langford et al. assessed bacterial coinfections during and after a COVID-19 diagnosis, identifying a primary coinfection prevalence of 3.5% (95% CI 0.4–6.7%) and a secondary (hospital-acquired) coinfection prevalence of 14.3% [10, 11]. The high coinfection rate in our study among kidney transplant patients post-COVID-19 can be attributed to the aggressive and timely implementation of bronchoscopies and bronchoalveolar lavage mNGS. Traditional pathogen testing methods, including microscopic examinations, cultures, serological antigen-antibody tests, and PCR, show significantly lower positive rates than mNGS, especially when determining coinfections with viruses and fungi. This aligns with the findings of Zhang’s research [12]. Traditional microbiological testing methods are technically mature and highly reliable with vast clinical applications. mNGS technology theoretically offers nonpresumptive, one-time testing for unknown pathogens with a broader detection scope. They recommend considering mNGS to identify pathogens, especially for immunosuppressed hosts suspected of having lower respiratory infections, suggesting causes other than common community-acquired pneumonia (CAP) pathogens [13, 14].

Currently, anti-coronavirus drugs (such as Nirmatrelvir/Ritonavir and Molnupiravir) are indicated for high-risk groups infected with coronavirus within 5 days of onset, which can reduce the rate of severe cases. Radcliffe C and others reported that Molnupiravir can reduce the severity and mortality rate in postkidney transplant patients infected with coronavirus [15]. In our study, the rates of disease exacerbation in the nonantiviral and antiviral drug groups were 22.2% and 14.3%, respectively. The rate of disease exacerbation in the antiviral drug group was lower than that in the nonantiviral group, but without statistic difference. (*P* > 0.05). Ritonavir has a strong drug-drug interaction with CYP3A-dependent drugs. Immunosuppressive drugs such as calcineurin inhibitors (e.g., tacrolimus and cyclosporine) and mammalian target of rapamycin (mTOR) inhibitors rely heavily on CYP3A metabolism, which are important drugs in KTRs. KTRs are particularly vulnerable to COVID-19 morbidity and mortality due to immunosuppression, comorbidities [16, 17].

In the American Society of Transplantation’s statement on oral antiviral therapy for COVID-19 in organ transplant recipients,Nirmatrelvir/Ritonavir poses significant challenges for use in many transplant patients. This is due to notable drug interactions and the complexities associated with therapeutic drug monitoring, particularly for outpatients actively infected with COVID-19. Molnupiravir, on the other hand, has shown relatively low efficacy and has not yet been evaluated in the context of transplant recipients [18].

Our analysis observed a lower rate of disease exacerbation among patients treated with antiviral drugs compared to those in the non-antiviral group (22.2% vs. 14.3%); however, this difference was not statistically significant. The small sample size in our research introduces a limitation that cannot be overlooked. As a result, it is imperative that further evaluations be conducted to ascertain the efficacy and safety of Oral Antiviral Therapy for COVID-19, particularly within the cohort of transplant recipients. During the follow-up study, the all-cause rehospitalization rate was 21.4%, with half of the cases being opportunistic lung infections (Pneumocystis jirovecii pneumonia). Risk factors for Pneumocystis jirovecii infection include the use of immunosuppressive agents, cancer, solid organ and stem cell transplantation, autoimmune diseases, inflammatory diseases, and congenital immune deficiencies. In recent years, the SARS-CoV-2 pandemic has become a concern. SARS-CoV-2 can impair the host’s immune system, increasing the risk of bacterial and fungal coinfections, including Pneumocystis jirovecii [19]. Therefore, SARS-CoV-2 infection has also become a risk factor for Pneumocystis jirovecii infection. Therefore, in patients with COVID-19 postkidney transplantation, there should be increased emphasis on the preventive treatment of Pneumocystis jirovecii pneumonia.

There are some limitations in our study: firstly, the KTR patients admitted to hospital were included, but not mild outpatient patients, which means that patients who need hospitalization might lead to selection of severe infection probably secondary to an opportunistic infections. This study does not reflect the generel population of KTR infected with COVID19. Secondly, the sample size is small and it is a single centre study, we hope to increase the sample size and research centers in the future.

In summary, our study indicates that KTRs are at a heightened risk for coinfections with the SARS-CoV-2 Omicron variant. We emphasize the importance of proactive bronchoscopy and bronchoalveolar lavage for precise pathogen identification, which is crucial for accurate diagnosis and can significantly contribute to reducing mortality rates among this vulnerable population. Furthermore, our findings suggest that acute exacerbations of cardiac and renal dysfunction, along with a history of opportunistic infections, are potential risk factors that may predict the deterioration of COVID-19 conditions post-hospital admission. These insights underscore the need for vigilant monitoring and tailored treatment strategies for KTRs to improve clinical outcomes during the ongoing pandemic.”

## Data Availability

No, I don’t have any research data outside the submitted manuscript file. The datasets generated during and analyzed during the current study are available from Dr. Lu.
